# Multimodal Assessment of Precentral Anodal TDCS: Individual Rise in Supplementary Motor Activity Scales With Increase in Corticospinal Excitability

**DOI:** 10.3389/fnhum.2021.639274

**Published:** 2021-02-25

**Authors:** Anke Ninija Karabanov, Keiichiro Shindo, Yuko Shindo, Estelle Raffin, Hartwig Roman Siebner

**Affiliations:** ^1^Danish Research Centre for Magnetic Resonance, Centre for Functional and Diagnostic Imaging and Research, Copenhagen University Hospital Hvidovre, Hvidovre, Denmark; ^2^Department of Nutrition, Exercise and Sports, University of Copenhagen, Copenhagen, Denmark; ^3^Department of Rehabilitation Medicine, Keio University School of Medicine, Shinjyuku-ku, Japan; ^4^Center for Neuroprosthetics and Brain Mind Institute, Swiss Federal Institute of Technology, Geneva, Switzerland; ^5^Institute for Clinical Medicine, University of Copenhagen, Copenhagen, Denmark; ^6^Department of Neurology, Copenhagen University Hospital Bispebjerg, Copenhagen, Denmark

**Keywords:** functional magnetic resonance imaging (fMRI), inter-individual variability, motor evoked potentials, primary motor cortex (M1), supplementary motor area (SMA), transcranial direct current stimulation (tDCS), non-invasive brain stimulation, transcrancial magnetic stimulation (TMS)

## Abstract

**Background:**

Transcranial direct current stimulation (TDCS) targeting the primary motor hand area (M1-HAND) may induce lasting shifts in corticospinal excitability, but after-effects show substantial inter-individual variability. Functional magnetic resonance imaging (fMRI) can probe after-effects of TDCS on regional neural activity on a whole-brain level.

**Objective:**

Using a double-blinded cross-over design, we investigated whether the individual change in corticospinal excitability after TDCS of M1-HAND is associated with changes in task-related regional activity in cortical motor areas.

**Methods:**

Seventeen healthy volunteers (10 women) received 20 min of real (0.75 mA) or sham TDCS on separate days in randomized order. Real and sham TDCS used the classic bipolar set-up with the anode placed over right M1-HAND. Before and after each TDCS session, we recorded motor evoked potentials (MEP) from the relaxed left first dorsal interosseus muscle after single-pulse transcranial magnetic stimulation(TMS) of left M1-HAND and performed whole-brain fMRI at 3 Tesla while participants completed a visuomotor tracking task with their left hand. We also assessed the difference in MEP latency when applying anterior-posterior and latero-medial TMS pulses to the precentral hand knob (AP-LM MEP latency).

**Results:**

Real TDCS had no consistent aftereffects on mean MEP amplitude, task-related activity or motor performance. Individual changes in MEP amplitude, measured directly after real TDCS showed a positive linear relationship with individual changes in task-related activity in the supplementary motor area and AP-LM MEP latency.

**Conclusion:**

Functional aftereffects of classical bipolar anodal TDCS of M1-HAND on the motor system vary substantially across individuals. Physiological features upstream from the primary motor cortex may determine how anodal TDCS changes corticospinal excitability.

## Introduction

Transcranial Direct Current Stimulation (TDCS) can non-invasively induce plasticity in the human brain by de- or hyperpolarizing neuronal membranes through the application of weak direct, electrical current. TDCS-induced plasticity is often demonstrated by bi-directional, polarity-specific effects on corticospinal excitability (Nitsche and Paulus, 2011). Using the amplitude of the motor evoked potential (MEP) as a measure of corticospinal excitability, many studies have demonstrated that corticospinal excitability increases when the anodal electrode (anodal TDCS) is placed over the primary motor hand area (M1-HAND) while it decreases when the cathodal electrode (cathodal TDCS) is placed over M1-HAND (Nitsche and Paulus, 2000; Liebetanz et al., 2002). Even though TDCS induced MEP changes have been replicated various times (for review Nitsche and Paulus, 2011), many recent reports, including a large double-blind, placebo-controlled trial, did not show significant effects of anodal TDCS on corticospinal excitability. These recent studies consistently found that the individual change in MEP amplitude was highly variable (Horvath et al., 2014; Lopez-Alonso et al., 2014; Wiethoff et al., 2014; Chew et al., 2015; Strube et al., 2016; Ammann et al., 2017; Lefebvre et al., 2019; Jonker et al., 2020). The number of participants displaying the “classical” anodal TDCS-induced increase ranging only between 30 and 50%, while the other participants showed no or the opposite effect (Lopez-Alonso et al., 2014; Wiethoff et al., 2014). The large variability in response patterns illustrates the need for a better understanding of the neurophysiological mechanisms that drive the changes in corticospinal excitability as well as the need to identify clinically applicable markers that can predict the individual response to TDCS in order to individualize stimulation (Karabanov et al., 2016).

Neuroimaging techniques like functional magnetic resonance imaging (fMRI) or (15)O-water positron emission tomography [(15)O-PET] can investigate the effects of non-invasive brain stimulation (NTBS), by using cerebral blood flow as a proxy for neural activity (Karabanov and Siebner, in press). Early investigations in the neurovascular response to brain stimulation demonstrated that changes are not restricted to the target site but that M1 stimulation affects activity and connectivity in a network of sensorimotor areas, most prominently the premotor cortex (PMC) and the supplementary motor area (SMA; Siebner et al., 2001; Lee et al., 2003). Early (15)O-PET studies used repetitive transcranial magnetic stimulation (TMS) to induce plasticity in M1-HAND, but more recent work using TDCS in combination with fMRI has shown similar effects: Anodal TDCS over M1-HAND modulates activity in M1 and SMA when applied at rest (Jang et al., 2009; Stagg et al., 2009) or when given during a motor task (Antal et al., 2011; Kwon and Park, 2011) and can impact functional coupling between the target region and remote network nodes (Antonenko et al., 2018). However, it is unclear whether the strength of TDCS-induced modulations in sensorimotor areas determines the individual change in corticospinal excitability measured by the MEP.

Improving the individual response to TDCS is important as TDCS-induced plasticity also modulates performance (Nitsche and Paulus, 2011): Several studies suggest that anodal TDCS of M1-HAND during motor training can improve training outcome (Reis and Fritsch, 2011) and TDCS is increasingly used to augment motor training (Buch et al., 2017). However, also performance improvements are reported to be highly variable (Ammann et al., 2016), limiting the use of TDCS as a tool in motor rehabilitation and highlighting the need for markers that can explain variability and guide personalization of stimulation protocols.

Several studies have identified physiological factors that influence variability of NTBS effects (Ridding and Ziemann, 2010; Guerra et al., 2020). One intriguing marker, that also implicates the responsiveness of areas upstream of M1-HAND in mediating corticospinal excitability changes after TDCS can be derived with single-pulse TMS of M1-HAND. It was shown that the latency difference between MEPs induced by anterior-posterior (AP) and lateral-medial (LM) current directions (i.e., AP-LM MEP latency), may predict the individual change in corticospinal excitability following TDCS (Wiethoff et al., 2014; McCambridge et al., 2015). While it was initially thought to reflect individual differences in inter-neuronal networks within M1-HAND (Hamada et al., 2013), the AP-LM MEP latency difference may alternatively reflect the preferential responsiveness of different parts of the precentral gyrus to transcranial electrical stimulation: Long AP-latencies indicate that AP-TMS targets more rostral parts of the precentral crown that are more upstream to M1-HAND (Aberra et al., 2020; Siebner, 2020). Conversely, short LM-latencies indicate that LM-TMS targets deeper parts of the precentral wall close to M1-HAND. Therefore, the latency difference between MEPs evoked by AP-TMS vs LM-TMS can be considered a physiological marker of individual microstructural properties of the precentral gyrus and their susceptibility to transcranial electrical stimulation.

Using a double-blinded placebo-controlled study design, we prospectively assessed the aftereffects of bipolar anodal TDCS targeting right M1-HAND on corticospinal excitability and task-related activation of frontal motor cortex. We complemented this interventional approach with singe-pulse TMS of M1-HAND to determine the individual AP-LM difference in MEP latency. This put us in a position to test whether TDCS-related changes in cortical motor activity in a broader set of sensory-motor areas scaled with the aftereffects of anodal TDCS on corticospinal excitability, accounting for interindividual differences in the susceptibility of the precentral gyrus to transcranial electrical stimulation.

## Materials and Methods

### Subjects

Twenty healthy volunteers were recruited via an advertisement posted on an open-access website for subject recruitment^1^ and completed both experimental sessions. All participants (10 women) were consistently right-handed (85.5 ± 15.3 points on Edenborough handedness Scale), non-smokers (Grundey et al., 2012) and had no history of previous neurological or psychiatric illness and no contraindication to NTBS or magnetic resonance imaging (MRI; Oldfield, 1971). The age ranged between 20 and 35 years (mean age. 25.0 ± 3.7). All participants gave informed consent for the purpose and procedures of the study. The study was conducted in accordance with the Declaration of Helsinki and approved by the Research Ethics Committees of the Capital Region (H-2-2013-040). Three participants were excluded because of missing data in the sham TDCS session. Another participant was excluded from fMRI analyses because of inability to correctly perform the behavioral task. Seventeen participants were included in the analyses of the MEP data and 16 participants were included in the analysis that included fMRI data.

### Experimental Procedures

**FIGURE 1:**
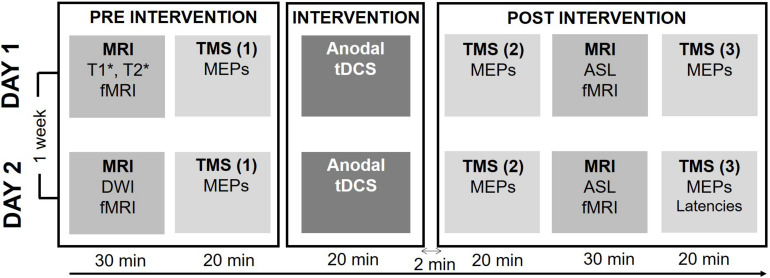
Experimental procedure. Each session started by a baseline measure consisting of a structural and functional MRI (fMRI) exam and baseline physiological measures of corticospinal excitability. Baseline behavioral measures of motor performance during a visuomotor tracking task were recorded during the fMRI sequence. After baseline measures, 20 min of either active or sham TDCS was applied. Directly after the intervention corticospinal excitability was reassessed, followed by the post-intervention run of the fMRI, Arterial Spin Labeling (ALS) and Resting-state fMRI (rs-fMRI) sequences. The post-intervention was concluded by the second measure of corticospinal excitability. In the second session, the individual latency profile was assessed by measuring the MEP latency following stimulation with different coil orientations.

Figure 1 provides a synopsis of the experimental procedures. Using a double-blinded cross-over design, we investigated how individual TDCS-induced changes in corticospinal excitability, as reflected by MEP amplitude evoked by single-pulse TMS, are associated with individual changes in regional cortical activity, as reflected by task-related BOLD-fMRI. Each participant received 20 min of real (0.75 mA) or sham (0 mA) TDCS on separate days in randomized order at least a week apart. Real and sham TDCS used the classic bipolar set-up with the anode placed over the right primary motor hand area (M1-HAND) and the cathode over the left supraorbital region. To avoid circadian fluctuations within participants both sessions were scheduled at the same time of day (Ridding and Ziemann, 2010).

Each experimental session started with a BOLD-fMRI run during which participants performed a visuospatial tracking (Raffin and Siebner, 2019). After baseline BOLD-fMRI, participants were moved out of the scanner and were placed in a comfortable chair in a laboratory adjacent to the MR-scanner, where MEPs were recorded to obtain a baseline measure of corticospinal excitability. Participants remained seated and received 20 minutes of real or sham TDCS. Immediately after the end of the TDCS-intervention corticospinal excitability was reassessed (T0) followed by task-related BOLD-fMRI using the same fMRI sequence and visuomotor task as at baseline. Thereafter, we measured brain perfusion using Arterial Spin Labeling (ASL) and performed resting-state fMRI (rs-fMRI). Sixty minutes after the TDCS intervention corticospinal excitability was reassessed (T60). At the end of the second and final session, we performed a comparison of MEP latencies in response to single-pulse TMS evoking an anterior-to-posterior or a medial-to-lateral current in the precentral gyrus using different coil orientations. During all TMS and TDCS procedures the participants were asked to remain seated comfortably, with resting hands and open eyes.

### MRI Measurements

Magnetic resonance imaging was performed using a Phillips 3 Tesla MR Achieva scanner (Philips Healthcare, The Netherland). BOLD signal during a visuomotor tracking task was assessed by a 10-min EPI-sequence (TR/TE = 1580/30 ms, field of view (FOV) 200 × 212 × 90, voxel size = 2.94 × 2.94 × 3mm, flip angle = 71°, number of slices = 30, no slice gap). The baseline scan in Session1 also included a high-resolution structural T1- and T2-weighted brain scan, which was used for neuronavigation of TMS. During the baseline scan of session 2, these scans were exchanged with a Diffusion Weighted MRI scan. Post-intervention MRI scans included, besides the EPI-sequence during visuomotor tracking, a resting-state fMRI (rs-fMRI) and a perfusion scan using ASL. The diffusion MRI, rs-fMRI, and ASL measurements were not included in this manuscript.

### Visuomotor Tracking During fMRI

The study was designed to delineate whether TDCS of right M1-HAND would produce lasting changes in task-related activity in the motor system. Therefore, participants performed a visuomotor tracking task during the fMRI session (Raffin and Siebner, 2019). The task was chosen because visuomotor tracking reliably activates the motor network including the SMA and the PMC (Ogawa et al., 2007) and required the participants to follow a continuously moving target line using an fMRI-compatible joystick (Hybridmojo, San Mateo, CA, United States). The joystick was operated by the left index finger and modified only to allow horizontal movements. The subjects’ left hand was placed palm down on the joystick using a foam wrist support to ensure that they had full index finger abduction and adduction range of motion. The joystick was attached to the subject’s left forearm such that they could only to move their left index to manipulate the joystick, with the remaining part of the arm completely still. The voltage signal representing joint motion was sent to a computer (Dell Computer Company, Round Rock, TX, United States) through an analog-to-digital converter that sampled the signal at 60 Hz. The peak of the target waveform was set at 85% of the standard range of motion (with 100% defined as a full extension), and the lower peak of the wave was set at 15% of the standard range of motion (with 0% defined as a full flexion). Thus, the upper and lower peaks of the target were within each subject’s range of motion. Before entering the scanner, the subjects were familiarized with the task and had the chance to practice the task for a few minutes.

Each fMRI run consisted of 30 blocks (block length approx. 20 s) during which, a target line continuously moved in the middle of the screen. Each block was preceded by a 2 to 4-s baseline with the target line being at a start position. Three different conditions were randomly alternating (resulted in from 8 to 12 blocks per condition): During *complex tracking*, the target line represented an unpredictable pattern that participants had to track using the joystick. During *simple tracking*, subjects had to track a highly predictable pattern. During *visual tracking*, participants had only to visually follow the target line. The line length was equal between conditions. The task was implemented in PsychoPy 2 (Version 1.8) (Peirce, 2008) and displayed on a 17-inch monitor with a resolution of 1280 × 1024 pixel situated at the end of the MRI tunnel that subjects viewed through a 45° oriented mirror placed above the eyes.

### Neurophysiological Measures

Transcranial magnetic stimulation measures were collected using a MagVenture MagPro R30 Stimulator (MagVentureA/S, Farum, Denmark) connected to a MC-B70 coil (MagVenture A/S, Farum, Denmark). TMS pulses were monophasic, induced a P-A current direction in the brain and were given with an inter-pulse interval of 0.2Hz. Correct positioning was continuously mirrored using a stereotaxic frameless neuronavigation system (LOCALITE GmbH, Sankt Augustin, Germany). Corticospinal excitability was evaluated before TDCS intervention (baseline), and 2 min and 1 h after the intervention (T0 and T60). Corticospinal excitability was measured by recording MEP amplitudes in response to an individually constant stimulation intensity (MEP amplitude): At the beginning of the experiment the individual stimulator intensity was set to evoke MEPs with a mean amplitude of 0.5 mV while at rest (Threshold_0.5_). Baseline recordings started with the identification of the motor hotspot for the left first dorsal interosseus (FDI) muscle. The motor hot spot was marked for online monitoring using neuronavigation and for re-identification of the hotspot during post-intervention recordings. In all sessions (pre and post) the Threshold_0.5_ was determined using the adaptive, parameter estimation by sequential testing method (adaptive PEST) (Awiszus, 2003; Karabanov et al., 2015). The initial output intensity for Threshold_0.5_ was used during both pre- and post-intervention measures to collect 20 MEPs. We also measured corticomotor latency of the MEP at the end of the experimental session at the second day (Hamada et al., 2013). To determine corticomotor MEP latency, we applied a single monophasic TMS pulse at motor hot spot. Stimulus intensity was adjusted to evoke a mean MEP amplitude of approximately 0.5 mV in the FDI muscle. MEPs were evoked with single monophasic TMS pulses inducing either a posterior-to-anterior (PA), anterior-to-posterior (AP), or latero-to-medial (LM) current direction in the precentral gyrus, while participants maintained a tonic contraction of the FDI muscle at 10% of maximal force level. To evaluate inter-individual differences in MEP latency, 20 MEPs were recorded for each coil orientation in a randomized order.

### TDCS Interventions

Direct current was generated by a DC-stimulator (NeuroCohn, GmbH, Ilmenau, Germany) via a pair of electrodes prepared with Ten20 Conductive Paste (Weaver and Company, CO, United States). The anodal electrode (3 cm × 4 cm) was placed over right M1-HAND with its center corresponding to the motor hot spot of the left FDI muscle. The motor hot spot was also marked on the scalp with the help of an individual anatomical MR scan of the whole brain and stereotaxic frameless neuronavigation. The cathodal electrode (5 cm × 7 cm) was attached to the left forehead above the orbit. Anodal TDCS was applied with an intensity of 0.75 mA for 20 min. We chose a small anodal electrode to be able to stimulate M1-HAND more focally, the relatively low current intensity was chosen to match the mA/cm^2^ current density usually achieved by the conventional 5 cm × 7 cm electrode at an intensity of 2 mV (0.625 mA/cm^2^ at 0.75 mV with 12 cm^2^ compared to 0.57 mA/cm^2^ at2 mA with 35 cm^2^).

**FIGURE 2:**
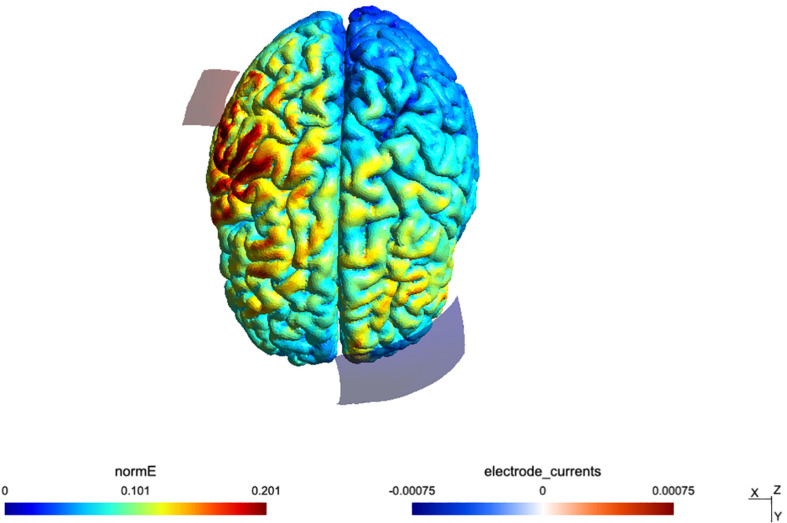
Simulation of the TDCS electric field for the montage, done using SimNIBS 2.1 and the included “Ernie” example dataset.

The fade-in fade-out period lasted 15 s. Sham TDCS consisted of the fade-in and fade-out phases only without any constant stimulation in between. A visualization of the applied montage and a calculation of the induced electric field was conducted with SimNIBS software (Thielscher et al., 2015) and a mean map of the electrical field distribution can be seen in Figure 2. After each interventional session, participants completed a questionnaire about TDCS-induced sensory effects (Brunoni et al., 2011). The study was double blinded since both, the participants and the examiner, responsible for the pre-post measures (MRI, TMS) were not aware of the type of stimulation in each session (sham or active).

### Statistical Analysis

#### Corticospinal Excitability

The mean MEP amplitude of the left FDI muscle was used as index of corticospinal excitability. Baseline MEP_PreSham_ and MEP_PreTDCS_ amplitudes were compared using a paired *t*-test to test whether corticospinal excitability was matched before the sham and real TDCS. For further investigation of TDCS-induced effects, the mean MEP_Post_ amplitudes were normalized to the pre-TDCS amplitudes of the same session by dividing MEP_Post_ by MEP_Pre_. The normalized MEPs of post1 and post2 measurements were entered as dependent variable in a two-way repeated measure analysis of variance (ANOVA) to investigate the effects of “Stimulation” (sham/TDCS) and Time (Post1/Post2).

#### MEP Latency

To investigate potential effects of TDCS on the MEP latency, two examiners independently measured the shortest latency of the superimposed MEP waveforms for each separate coil orientation (Hamada et al., 2013). We computed the Pearson correlation coefficient to test for correlations between the normalized amplitudes of MEP_Post1_ and MEP_Post2_ and the orientation-related differences in MEP latency (PA-LM and AP-LM orientations).

#### Behavioral Data

The absolute mean of the tracking error was calculated for both movement conditions (complex tracking versus simple tracking). If (*xt*) is the instantaneous horizontal coordinate of the target line and (*xj*) the instantaneous horizontal coordinate of the joystick, then the instantaneous error at that time point was defined as:

$$Instantaneouserror=\sqrt{{\left(xt-xj\right)}^{2}}-r$$

where *r* is the radius of the circle around the joystick (i.e the tolerance area). The improvement (Errror_Imp_) across the pre- and post-intervention scan was calculated by subtracting the absolute mean Error_%Post_ from the absolute mean Error_pre_ for each participant. A two-factorial ANOVA with the dependent factor Errror_Imp_ and the independent factors “Stimulation” (real TDCS/sham TDCS) and “Task” (complex/simple), was calculated to focus on potential TDCS-induced performance changes. Effects were considered significant at *p* < 0.05.

#### Analysis of Task-Based fMRI Data

Functional magnetic resonance imaging data analysis was performed using SPM8 (Wellcome Department of Cognitive Neurology, London, UK) and MATLAB R2012a (Mathworks, Natick, MA, United States). Data from each participant were motion-corrected, realigned and smoothed with an 8-mm isotropic Gaussian kernel. At the first level, images related to the amplitude of the hemodynamic response were entered into the full factorial ANOVA model in each subject modeling “Stimulation,” “Task,” and “Time.” At the second level, contrast images were collected into one sample *t*-test. To investigate correlations between TDCS-induced effects on movement-related BOLD activity and TDCS-induced effects on corticospinal excitability, the contrast images “*simple tracking and complex tracking* versus rest” were entered into an SPM regression with the normalized MEP as covariates (i.e., independent variable). A statistical threshold of *p* < 0.05 (FWE corrected at the cluster level) was used to identify significantly activated regions on the group level, applying a non-corrected cluster extent threshold of *p* < 0.001). For nodes of the sensorimotor network known to be affected by TDCS (SMA, PMd, and M1-HAND) we constructed spherical volume-of-interest (VoI) with a 10 mm radius. The center of the spherical VoI matched peak coordinates was center coordinates based on task-based peak activations reported in a previous fMRI study (Lee et al., 2003). Small volume correction was applied for voxels within the VoIs.

#### Multiple Regression Analysis

We tested whether a combination of predictor variables (BOLD change in SMA and MEP latency difference depending on AP-LM current orientation) could predict TDCS-induced change in MEP amplitude. We computed a multiple regression analysis in which the normalized MEP at post1 was treated as dependent variable. The change in task-related BOLD signal in SMA and the AP-LM latency difference were entered as explanatory variables. The linear regression model was calculated in R and used the lm function (R Core Team, 2017). The relative importance of each predictor was determined using bootstrap confidence intervals for relative importance (function boot.relimp) (R Core Team, 2017).

#### Questionnaires

Feedback about the sensory side effects of real and sham TDCS stimulation was analyzed using a questionnaire (Brunoni et al., 2011). A Fisher’s exact test was performed to differences in questionnaire ratings between TDCS and sham sessions. Statistical analyses were performed with SPSS version 19.0, with exception of the Multiple Regression analysis.

## Results

Data from the post-stimulation questionnaire (see Appendix) indicated that participants could not distinguish between the sham and real TDCS intervention. There was no significant difference ratings of any item (*p* > 0.05, Fisher’s exact test).

### Corticospinal Excitability

**FIGURE 3:**
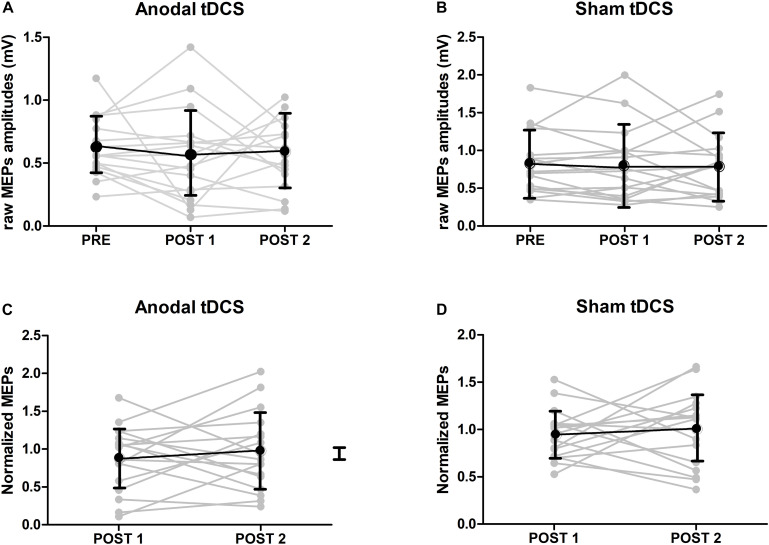
MEP results. **(A)** Raw amplitudes of MEPs after either anodal or sham TDCS (mean ± SE). At baseline, there was a significant difference in MEP amplitudes between TDCS and sham sessions (*p* < 0.05, paired t-test). **(B)** Group results of normalized amplitudes of MEPs after either anodal or sham TDCS (mean ± SE, *n* = 17). Normalized amplitudes were calculated by dividing the amplitudes of MEPs just after or 1 hour after TDCS by ones at baseline. No interaction could be detected.

The mean MEP amplitudes at baseline and after TDCS are shown in Figure 3. At baseline, there was a significant difference in MEP amplitudes between the real and sham TDCS sessions (*p* = 0.034, paired *t*-test. This difference was caused by higher baseline MEP amplitudes in the sham TDCS session Figure 3A. Using the non-normalized MEPs in a 3 × 2 ANOVA with the factors Stimulation (anodal/sham) and Time (pre/T0/T60) a main effect of Stimulation [*p* = 0.002, *F*(16) = 9.99] was detected, indicating a difference in MEP amplitude between the sham and real TDCS sessions but the ANOVA showed neither a significant main effect of Time [*p* = 0.32, F(16) = 0.99] nor an interaction between Time and Stimulation [*p* = 0.71, F(16) = 0.17]. To check if the baseline difference in MEP amplitude affected the results, we ran a post-hoc analysis where the same ANOVA was repeated after removing the three individuals with the highest MEP amplitudes during sham. This analysis (*N* = 14), confirmed that there was no significant effect of TDCS or Time x TDCS interaction when the baseline difference between groups was eliminated (all *p*-values >0.13). In an additional analysis (*N* = 17), we normalized post-TDCS MEP amplitudes at T0 and T60 to individual mean MEP amplitude at baseline. Normalized MEP amplitudes were entered in a 2 × 2 ANOVA with the factors Stimulation (anodal/sham) and Time (T0/T60). No main effect or interaction was detected by this analysis (*p* > 0.5 for all). The normalized group data are illustrated in Figure 3.

### Orientation Dependency of MEP Latency and TDCS Aftereffect on MEP Amplitudes

**FIGURE 4:**
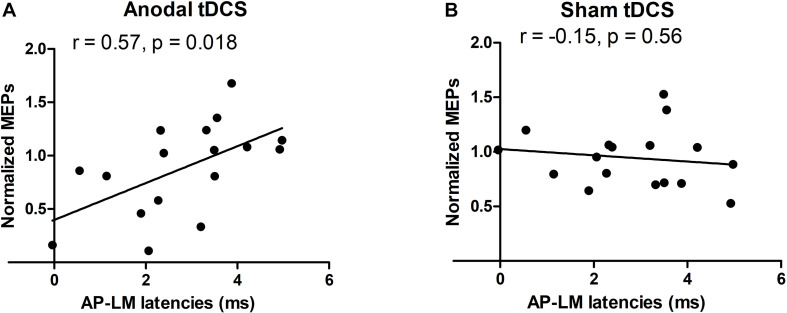
Relationships between the normalized TMS amplitudes and AP-LM latency. A positive correlation was found just after the anodal session **(A)**, but neither just after the sham session (B), nor 1 h after each stimulation. For the *y*-axis a value of 1 is equivalent to no change from baseline.

Mean corticomotor latencies of the MEPs were 22.1 ms (±1.7) for the PA current direction, 24.5 ms (±2.0) for the AP current direction and 21.6 ms (±1.8) for the LM current direction. There was a significant positive correlation between the individual difference between the MEP latency evoked with AP versus LM current orientation and the individual change in normalized MEP amplitude immediately after the anodal TDCS (post1) (*R* = 0.57, *p* = 0.018, Figure 4A). The larger the relative delay in MEP latency at AP versus LM current direction, the larger the individual increase in MEP amplitude after real anodal TDCS. No such relationship was found for the sham TDCS session (*R* = −0.15, *p* = 0.56, Figure 4B). There was no significant correlation between AP-LM latency and normalized amplitudes of MEPs one hour after either the anodal TDCS (*R* = 0.24, *p* = 0.36) or the sham (*R* = −0.15, *p* = 0.56). There was no significant correlation between PA-LM latencies and normalized amplitudes of MEPs just after the anodal TDCS (*R* = 0.26, *p* = 0.31) or the sham (*R* = 0.05, *p* = 0.86).

#### Task Performance During Visuomotor Tracking

We computed a two-factorial ANOVA to investigate the effects of sham and anodal TDCS on task performance. We found a main effect of Task [*F*(16) = 1.5396, *p* = 0.02] of the tracking error, showing better performance during the simple tracking condition. There was no main effect of Stimulation [*F*(16) = 1.53, *p* = 0.21] and no Task × Stimulation interaction [*F*(16) = 0.02, *p* = 0.88], indicating that TDCS did not modify visuomotor tracking performance.

#### Task-Based BOLD Signal Changes

**FIGURE 5:**
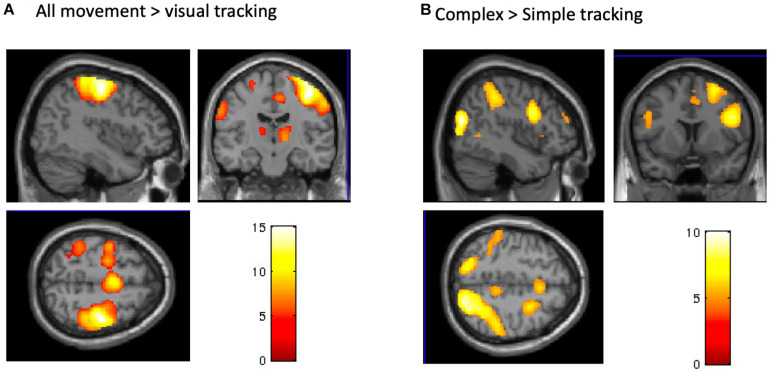
BOLD signals during the tracking task. **(A)** A conjunction analysis between complex and simple visuomotor tasks. The significant regions were the right precentral gyrus, bilateral SMA, contralateral postcentral gyrus, and IPC and SPL (p < 0.05, FWE). **(B)** A contrast (complex > simple visuomotor tasks). The significant regions were the right middle occipital gyrus, bilateral SPL, right IPC and premotor cortex, and bilateral SMA (p < 0.05, FWE).

The post-TDCS fMRI session started on average 20 (±3.3) minutes after TDCS. The complex and simple visuomotor finger tracking tasks induced significant BOLD signal increases in the right precentral cortex and in a broad bilateral network, including the SMA, ventral premotor and parietal cortex (IPC) (FWE, *p* < 0.05), when compared to visual tracking alone (Table 1 and Figure 5A). The complex visuomotor tracking task induced more activation in the right middle occipital gyrus, bilateral parietal cortex, right dorsal PMC and SMA when compared to the simple task (FWE, *p* < 0.05) (Table 2 and Figure 5B. *Anodal TDCS* induced no significant change in task-related fMRI activity compared to sham TDCS. There was also no significant interaction between Stimulation and Task (at FWE < 0.05, whole brain or small volume corrected in VOIs).

**TABLE 1:** Task activations for the conjunction analysis between complex and simple task.

Coordinates			Brain region	Cluster size
x	y	z		
**42**	**−16**	56	**R primary motor cortex**	**6924**
44	**−**32	64	R superior parietal lobule	
0	0	54	R supplementary motor cortex	
44	**−**32	64	R superior parietal cortex	
**−52**	**−24**	**40**	**L inferior parietal lobule**	**1538**
**−**40	**−**36	48	L superior parietal lobule	
**−**34	**−**37	61	L primary sensory cortex	
**−58**	**6**	**32**	**L ventral premotor cortex**	**378**
**−**54	8	18	L inferior frontal gyrus	
**−2**	**−56**	**−2**	**L cerebellar vermis**	**32**
**14**	**−16**	**2**	**R thalamus**	**366**
**−12**	**−4**	**−14**	**L thalamus**	**160**
**60**	**10**	**28**	**R inferior frontal gyrus**	**268**
**−26**	**−2**	**2**	**L putamen**	**129**
**−44**	**−2**	**6**	**L Insula**	**57**

*Bold values: correction for multiple comparisons used the FWE method at a corrected p < 0.05.*

**TABLE 2:** Task activations when the complex motor task was compared to the simple motor task.

Coordinates			Brain region	Cluster size
x	y	z		
**44**	**−76**	**18**	**R superior parietal cortex**	**2018**
30	**−**70	44	R middle occipital cortex	
50	34	44	R inferior parietal cortex	647
**−50**	**−34**	**42**	**L inferior parietal cortex**	647
**46**	**8**	**28**	**R inferior frontal gyrus**	**243**
**−46**	**6**	**28**	**L inferior frontal gyrus**	**204**
30	6	60	R dorsal premotor cortex	955
**6**	**18**	**46**	**R supplementary motor cortex**	**468**

*Bold values: correction for multiple comparisons used the FWE method at a corrected p < 0.05. Cluster size is indicated in voxel.*

#### Relationship Between Corticospinal Excitability and BOLD Response

**FIGURE 6:**
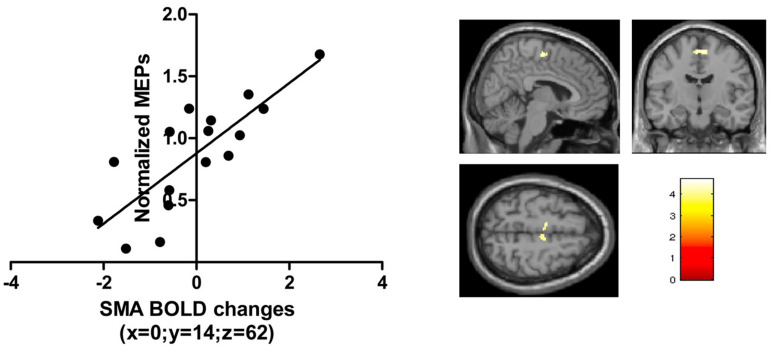
The *t*-scores in the contrast visuomotor tracking vs visual baseline correlated with the normalized TMS amplitudes just after anodal TDCS in a region in the bilateral SMA [peak activation [0, -14, 62]; *p* < 0.05 FWE; small volume correction; 10 mm sphere based on Lee et al. (2003)].

The normalized MEP immediately after real anodal TDCS (T0) was positively correlated with BOLD signal change during the visual-motor tracking task (complex and simple combined) after anodal TDCS. Significant correlations were found in the bilateral SMA (*x* = 6, *y* = −12, *z* = 60, kE 374, T score = 5.95, *p* = 0.014, FWE, small volume corrected, Figure 6). There were no significant correlations just after sham stimulation, or 1 h after anodal TDCS, or using the pre-TDCS MEP values or AP-LM latency difference of the MEPs.

#### Multiple Regression Analysis

In a follow-up analysis, we specified a multiple regression model combining explanatory variables (AP-LM MEP latency and BOLD signal change in SMA). The combined model explained 54% of the variance in normalized MEP amplitude immediately after real TDCS: (Multiple *R*^2^ = 0.54; *R*^2^-adjusted = 0.47; *p* = 0.005). Both explanatory variables had an independent predictive value (signal change in SMA; *p* = 0.043, AP-LM latency; *p* = 0.007) but the AP-LM latency had a higher relative importance (0.65) compared to the task-related BOLD change in the SMA (0.34) (R Core Team, 2017). Both explanatory variables had a variance inflation factor (VIF) of 1.00 indicating that collinearity was not an issue in the model.

## Discussion

In this double-blinded, placebo-controlled study, we prospectively assessed the functional aftereffects of low-intensity anodal TDCS over M1-HAND on corticospinal excitability at rest as well as functional cortical activation and performance during visuomotor tracking. At a current intensity of 0.75 mA, 20 min of anodal TDCS did not trigger a consistent modulation of corticospinal excitability, task-related cortical activity or motor performance on a group level. We found that the individual increase in MEP amplitude shortly after anodal TDCS correlated positively with a stronger functional recruitment of SMA during the visuomotor task shortly after TDCS. This correlation was not present after sham stimulation.

Our null finding that anodal TDCS did not have a consistent group effect on corticospinal excitability fits with other studies reporting high variability and a high non-responder rate (Horvath et al., 2014; Lopez-Alonso et al., 2014; Ammann et al., 2017). Together, this recent work suggests that current intensities up to 2 mA may be below the intensity needed to efficiently affect intrinsic neural spiking activity in the cortical target, at least when using the classical bipolar M1-supraorbital montage (Voroslakos et al., 2018). The positive linear relationship between the TDCS-induced increase in task-related SMA activity and TDCS-induced MEP amplitude change suggests that low-intensity TDCS may influence the cortical motor system upstream from M1-HAND.

Several studies have demonstrated that stimulation-induced alterations in corticospinal excitability are associated with more widespread changes in the sensorimotor network (Lang et al., 2005; Jang et al., 2009; Stagg et al., 2009; Gao et al., 2020). Non-invasive transcranial stimulation of the M1-HAND results in stronger functional coupling between the SMA and the sensorimotor cortex (Lee et al., 2003) and leads to increased regional activity in the SMA (Jang et al., 2009; Stagg et al., 2009; R Core Team, 2017; Voroslakos et al., 2018). Our results are in good agreement with these studies, showing a linear relationship between the TDCS effects on corticospinal excitability and changes in task-related activity of the SMA. The results may be accounted for by two mechanisms. On the one hand, the TDCS effect on corticospinal excitability may have triggered a compensatory increase in SMA activity in order to maintain overall network balance despite a change in the corticospinal output function. On the other hand, the TDCS-induced change in corticospinal excitability may have been mediated by an upstream modulation of SMA activation. While the present study cannot differentiate between the two mechanisms, our findings add evidence to a relevant role of the SMA in mediating the neuromodulatory effects of classical bipolar TDCS with the anode placed over the M1-HAND.

Our multiple regression analysis indicated that the AP-LM latency differences had a higher predictive power than the task related SMA activity even though both variables uniquely contributed to explain inter-subject variations in corticospinal facilitation after anodal TDCS. The electrophysiological results confirm the well-known dependency of MEP latencies on the TMS-induced current orientation in the precentral gyrus (Hamada et al., 2013). The larger the latency difference between AP and LM oriented TMS, the more rostrally the AP-TMS stimulus excites cortical neurons in the precentral crown and the more caudally the LM-TMS stimulus excites cortical neurons in the depth of the precentral wall (Hamada et al., 2013; Siebner, 2020). Several previous studies have investigated if individual differences in orientation-dependent MEP latencies relate to TDCS-induced aftereffects and our work is the third study showing a linear relationship between orientation dependency of AP-LM MEP latency and anodal TDCS aftereffects on corticospinal excitability (Wiethoff et al., 2014; Davidson et al., 2016). However, while previous studies reported negative correlations our study found a positive relationship between the orientation-depended latency difference and MEP amplitudes. One possible reason of the discrepancy is the difference in current intensity between studies: Studies that report negative correlations used stronger currents (2mA) than the present study and it may be that that stimulation intensities interact with the AP-LM latency to differentially determine the efficacy to induce LTP or LTD-like aftereffects. Using transcranial magnetic stimulation (TMS) to stimulate the M1-HAND, Hamada and colleagues found that a large AP-LM MEP latency favors a “canonic” plasticity response, being associated with a large MEP increase after a “facilitatory” TMS protocol (intermittent theta burst stimulation) and a larger MEP decrease after an “inhibitory” TMS protocol (continuous theta burst stimulation) (Hamada et al., 2013). This shows that the sign of the linear relationship between AP-LM MEP latency and the stimulation-induced MEP change may flip when changing a variable of the interventional protocol such as the temporal pattern of stimulation, but possibly also the intensity of stimulation. Hence, the impact of AP-LM latency may “flip” when the intensity of anodal TDCS is increased.

The timing of measurements relative to the administration of TDCS may also have contributed to the discrepant findings regarding the relationship between AP-LM MEP latency and the TDCS-induced change in MEP amplitude. The two studies that reported a negative relationship did measure the AP-LM latencies prior to the TDCS-intervention, whereas our study found a positive relationship and AP-LM latencies were measured at the very end of the second testing day. Indeed, it has been shown that the AP-LM latency itself can be modulated by plasticity-inducing NTBS interventions (Volz et al., 2019). Although the timing of latency measurement relative to TDCS may play a role in determining the relationship between AP-LM MEP latency and TDCS-induced MEP changes, we consider it unlikely that latency measures were significantly influenced by TDCS in this study. We measured latencies approximately one hour after the end of stimulation to minimize the influence of TDCS aftereffects. Further, measurements were performed on the second experimental day, on which half of the participants received sham TDCS. However, more research is needed to clarify how stimulation variables and the relative timing between stimulation and measurements influence the aftereffects of TDCS.

The method to assess the difference in AP-LM MEP latency may also be relevant. While we used the shortest MEP latency out of 20 superimposed MEP waveforms for each separate coil orientation to determine the AP and LM latency, others calculate the AP and LM latency based on an averaging procedure that takes into account all MEPs (Jonker et al., 2020). Using the latter procedure, a recent larger double-blind trial did not find AP-LM MEP latency differences to reliably predict TDCS-induced aftereffects when using a stimulation intensity of 2 mA (Jonker et al., 2020). Together, the existing data on, the predictive value of the individual difference in AP-LM MEP latency on TDCS-related aftereffects on corticomotor excitability are highly interesting, but more research on the impact of methodological factors and specific features of the TDCS protocol, such as intensity or montage, is needed to assess the usefulness of the AP-LM MEP latency as predictive variable in future TDCS studies.

Anodal TDCS over M1-HAND did not affect performance during the visuomotor tracking task. Previous literature on the effects of TDCS on motor performance has suggested that behavioral effects are most prominent when TDCS is applied concurrently with the training task or when the motor task and stimulation are interleaved (Reis and Fritsch, 2011; Buch et al., 2017). This may explain why we were not able to show a measurable effect of anodal TDCS on motor performance. Alternatively, continuous visuomotor tracking may be a motor skill that may not benefit from anodal TDCS or would require a higher current intensity to show consistent effects of TDCS at the behavioral level.

A strength of this study is its double-blinded, placebo-controlled study design. MEP measurements are strongly dependent on the investigator holding the coil, even if neuro-navigation and other standard methods are applied. Knowledge about the session type might lead the experimenter to unconsciously influence study outcome and thereby artificially increasing the effect size and studies with a similarly rigorous design have also not shown effects of anodal TDCS on corticospinal excitability, even at significantly higher stimulation intensities (2 mA) (Jonker et al., 2020). A limitation of this study was that mean MEP amplitudes at pre-TDCS baseline were not matched between the real and sham TDCS conditions. This between-session difference emerged despite of our attempts to keep variability between sessions as small as possible by choosing a within-subject design, MRI-guided neuronavigation and controlling for circadian variations by scheduling both sessions at the same time of day. However, we don’t think that these differences in baseline MEP between sessions challenge the main conclusions of this study as a post-hoc analysis, in which the individuals that caused the baseline difference, were removed did not alter our conclusions.

## Conclusion

The after-effects of weak-current (0.75 mA) anodal TDCS stimulation targeting M1-HAND are highly variable, confirming several anodal TDCS studies, using the same electrode set-up but higher current intensities. Individual susceptibility to the neuromodulatory effects of TDCS on corticospinal excitability is likely to be determined by various physiological factors, including physiological properties of the precentral gyrus – as reflected by the orientation-dependent effect of single TMS on MEP latency and by the fact that the response pattern was predicted by individual differences in sensitivity to coil orientation. Further, the magnitude of TDCS-induced changes in corticospinal excitability correlated positively with the TDCS-induced increase in BOLD activity in the SMA. This linear relationship suggests that physiological features upstream from the primary motor cortex may mediate how anodal TDCS changes corticospinal excitability.

## Data Availability Statement

The raw data supporting the conclusions of this article will be made available by the authors, without undue reservation.

## Ethics Statement

The studies involving human participants were reviewed and approved by Research Ethics Committees of the Capital Region H-2-2013-040. The patients/participants provided their written informed consent to participate in this study.

## Author Contributions

AK, KS, ER, and HS designed the study. KS, YS, and AK collected the data. KS and AK analyzed the data. AK, KS, YS, ER, and HS wrote the manuscript. All authors contributed to the article and approved the submitted version.

## Conflict of Interest

HS has received honoraria as speaker from Sanofi Genzyme, Denmark and Novartis, Denmark, as consultant from Sanofi Genzyme, Denmark, Lophora, Denmark, and Lundbeck AS, Denmark, and as editor-in-chief (Neuroimage Clinical) and senior editor (NeuroImage) from Elsevier Publishers, Amsterdam, The Netherlands. He has received royalties as book editor from Springer Publishers, Stuttgart, Germany and from Gyldendal Publishers, Copenhagen, Denmark. The remaining authors declare that the research was conducted in the absence of any commercial or financial relationships that could be construed as a potential conflict of interest.

## Footnotes

1 www.forsogsperson.dk

## Appendix

### Appendix Side Effects of TDCS

**Table:** There was not a significant difference in the items rating two (mild) or more points on the questionnaire (*p* > 0.05, Fisher’s exact test).

	Anodal (*n* = 17)	Sham (*n* = 17)	Fisher’s exact test
Headache	3	2	1.000
Neck pain	2	2	1.000
Scalp pain	3	1	0.601
Tingling	5	10	0.166
Itching	7	5	0.721
Burning	9	11	0.728
Redness	2	0	0.485
Sleepness	11	9	0.728
Trouble concentraining	7	4	0.465
Acute mood change	0	0	**–**

## Abbreviations

AP: anterior-to-posterior (current direction)
BOLD: blood oxygenation level dependent
FDI: first dorsal interosseous
fMRI: functional magnetic resonance imaging
LM: latero-medial (current direction)
M1: primary motor cortex
M1-HAND: primary motor hand area
MEP: motor evoked potential
PA: posterior-to-anterior (current direction)
RMT: resting motor threshold
VoI: volume of interest
S1: primary sensory cortex
SMA: supplementary motor area
TDCS: transcranial direct current stimulation
TMS: transcranial magnetic stimulation
PEST: parameter Estimation by Sequential Testing.
